# GABBR2 as a Downstream Effector of the Androgen Receptor Induces Cisplatin Resistance in Bladder Cancer

**DOI:** 10.3390/ijms241813733

**Published:** 2023-09-06

**Authors:** Mohammad Amin Elahi Najafi, Masato Yasui, Yuki Teramoto, Tomoyuki Tatenuma, Guiyang Jiang, Hiroshi Miyamoto

**Affiliations:** 1 Department of Pathology and Laboratory Medicine, University of Rochester Medical Center, Rochester, NY 14642, USA; mohammadamin_elahinajafi@urmc.rochester.edu (M.A.E.N.); yabomabo@gmail.com (M.Y.); tera1980@kuhp.kyoto-u.ac.jp (Y.T.); tatenuma@yokohama-cu.ac.jp (T.T.); jianggy@hotmail.com (G.J.); 2 James P. Wilmot Cancer Institute, University of Rochester Medical Center, Rochester, NY 14642, USA; 3 Department of Urology, University of Rochester Medical Center, Rochester, NY 14642, USA

**Keywords:** androgen receptor, chemoresistance, cisplatin, GABBR2, urothelial cancer

## Abstract

The precise molecular mechanisms responsible for resistance to cisplatin-based chemotherapy in patients with bladder cancer remain elusive, while we have indicated that androgen receptor (AR) activity in urothelial cancer is associated with its sensitivity. Our DNA microarray analysis in control vs. AR-knockdown bladder cancer sublines suggested that the expression of a GABA B receptor GABBR2 and AR was correlated. The present study aimed to determine the functional role of GABBR2 in modulating cisplatin sensitivity in bladder cancer. AR knockdown and dihydrotestosterone treatment considerably reduced and induced, respectively, GABBR2 expression, and the effect of dihydrotestosterone was at least partially restored by an antiandrogen hydroxyflutamide. A chromatin immunoprecipitation assay further revealed the binding of AR to the promoter region of *GABBR2* in bladder cancer cells. Meanwhile, *GABBR2* expression was significantly elevated in a cisplatin-resistant bladder cancer subline, compared with control cells. In AR-positive bladder cancer cells, knockdown of GABBR2 or treatment with a selective GABA B receptor antagonist, CGP46381, considerably enhanced the cytotoxic activity of cisplatin. However, no additional effect of CGP46381 on cisplatin-induced growth suppression was seen in GABBR2-knockdown cells. Moreover, in the absence of cisplatin, CGP46381 treatment and GABBR2 knockdown showed no significant changes in cell proliferation or migration. These findings suggest that GABBR2 represents a key downstream effector of AR signaling in inducing resistance to cisplatin treatment. Accordingly, inhibition of GABBR2 has the potential of being a means of chemosensitization, especially in patients with AR/GABBR2-positive bladder cancer.

## 1. Introduction

Urothelial carcinoma in the urinary bladder has represented a commonly diagnosed malignancy, especially in men, and the global number of bladder cancer-related deaths has indeed increased from 165,100 in 2012 [1] to 212,536 in 2020 [2]. Particularly, muscle-invasive bladder cancer is often associated with metastatic disease and poor patient outcome (e.g., the overall 5-year survival rate of 8.3% [3]). Moreover, urothelial carcinoma also arises in the upper urinary tract but is much more often (e.g., 60% [4]) invasive at the initial diagnosis.

Despite the availability of novel therapeutic options, such as immunotherapy with checkpoint inhibitors, cisplatin (CDDP)-based chemotherapeutic regimens remain the first-line systemic treatment for locally advanced or metastatic urothelial carcinoma [5,6,7,8]. In addition, a meta-analysis involving 13,391 patients showed a significant overall survival benefit of neoadjuvant chemotherapy with CDDP-containing regimens prior to radical cystectomy [9]. Nonetheless, the response rate to CDDP therapy is not necessarily high (e.g., up to 60% [10,11,12]) in patients with bladder cancer. Accordingly, the development of not only novel anti-cancer agents but also strategies for chemosensitization of existing drugs should provide considerable improvement in oncologic outcomes of urothelial cancer.

The molecular mechanisms underlying CDDP resistance are still far from being fully understood [13,14,15,16,17,18]. Meanwhile, androgen-mediated androgen receptor (AR) signaling has been implicated in the promotion of urothelial tumorigenesis, which may be a reason for a substantially higher risk of bladder cancer development in men than in women, as well as urothelial tumor progression [19]. More fascinatingly, AR activity has also been associated with sensitivity to conventional nonsurgical treatments for bladder cancer [20], including CDDP-based chemotherapy [21,22,23]. Specifically, AR activation in bladder cancer cells has been shown to result in the induction of CDDP resistance, and AR knockdown or antiandrogen treatment thus enhances its sensitivity. Importantly, it remains to be further elucidated how the AR pathway modulates chemosensitivity in urothelial cancer. The present study aimed to explore whether gamma-aminobutyric acid (GABA) B receptor 2 (GABBR2), which belongs to the G protein-coupled receptor superfamily, functions as a downstream effector of AR and induces resistance to CDDP therapy in bladder cancer.

## 2. Results

### 2.1. AR Activity and GABBR2 Expression

**Figure 1:**
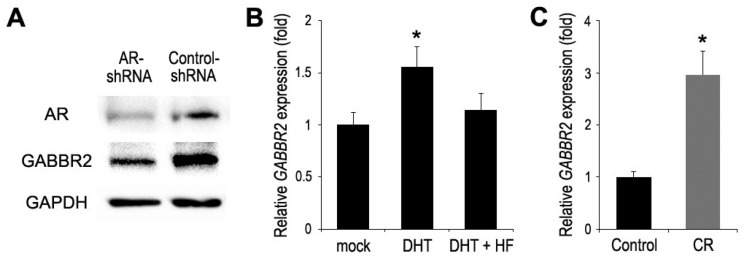
Associations between AR signaling and GABBR2 expression in bladder cancer cells. (**A**) Western blotting of AR and GABBR2 in UMUC3-AR-shRNA vs. UMUC3-control-shRNA. Real-time PCR of *GABBR2* in UMUC3 cultured for 24 h with ethanol (mock), 10 nM DHT, and/or 5 µM HF (**B**) or control UMUC3 vs. CDDP-resistant (CR) UMUC3 (**C**). The expression of *GABBR2* normalized to that of *GAPDH* and representing the mean (+SD) of at least three determinants is presented relative to that of mock treatment (**B**) or control subline (**C**). * *p* < 0.05 (vs. mock or control).

We had recently employed DNA microarray analysis in control AR-positive bladder cancer UMUC3 cells versus a subline of UMUC3 stably expressing AR-shRNA [24]. We then investigated whether the expression of 25 candidate genes was correlated with that of AR. We eventually selected one of the candidates, GABBR2, and confirmed that its protein expression was indeed downregulated in AR-knockdown cells, compared with control cells (Figure 1A). A quantitative PCR in UMUC3 cells further showed that androgen (i.e., dihydrotestosterone (DHT)) treatment induced *GABBR2* expression, which was at least partially blocked by antiandrogen (i.e., hydroxyflutamide (HF)) treatment (Figure 1B). Thus, GABBR2 expression was correlated with the expression and activity of AR in bladder cancer cells. Meanwhile, the level of *GABBR2* expression in a CDDP-resistant subline was significantly higher than that in control cells (Figure 1C).

**Figure 2:**
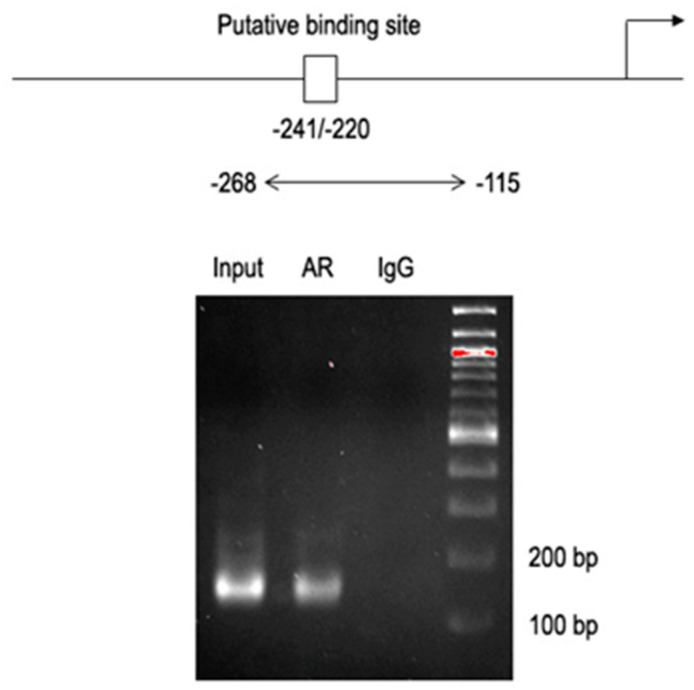
AR binding to the GABBR2 promoter in bladder cancer cells. The ChIP assay, using UMUC3 cell lysates immunoprecipitated with an anti-AR antibody (or IgG as a negative control). The DNA fragments were PCR amplified with a set of GABBR2 promoter-specific primers, and the PCR products were electrophoresed on 1% agarose gel. A fraction of the mixture of protein-DNA complex (i.e., 1% of total cross-linked, reserved chromatin prior to immunoprecipitation) was used as “input” DNA.

We next examined if AR could regulate the expression of GABBR2, using a chromatin immunoprecipitation (ChIP) assay (Figure 2). A bioinformatics-driven search detected a potential binding site of AR in the promoter region of the *GABBR2* gene. DNA fragments derived from UMUC3 cells immunoprecipitated with an anti-AR antibody were amplified by PCR with a set of GABBR2 promoter-specific primers. The PCR product was visualized from those precipitated by the AR antibody, but not control precipitations, indicating that AR could interact with the GABBR2 promoter.

### 2.2. Role of GABBR2 in Cell Growth

**Figure 3:**
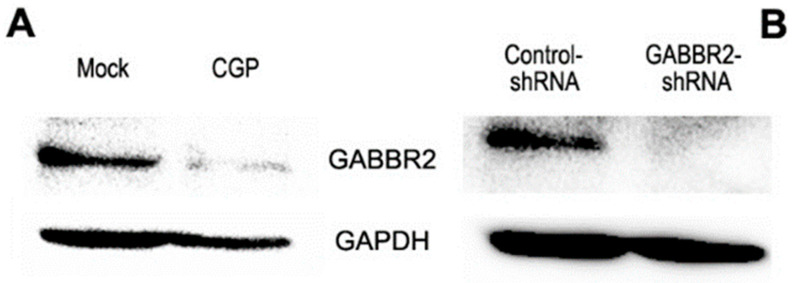
Effects of GABBR2 inhibitor or knockdown in bladder cancer cells. Western blotting of GABBR2 in UMUC3 cells cultured for 24 h with ethanol (mock) or CGP46381 (**A**) or UMUC3-control-shRNA vs. UMUC3-GABBR2-shRNA sublines (**B**).

**Figure 4:**
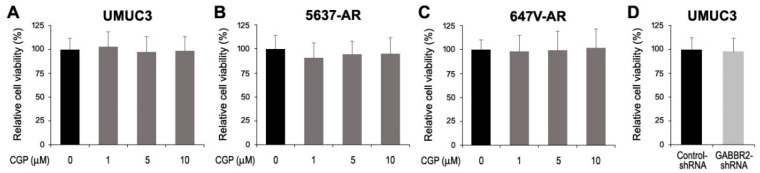
Effects of GABBR2 inhibitor or knockdown on the viability of bladder cancer cells. The MTT assay in UMUC3 (**A**), 5637-AR (**B**), or 647V-AR (**C**) cultured in the absence or presence of various doses of CGP46381 or UMUC3-control-shRNA vs. UMUC3-GABBR2-shRNA (**D**) for 48 h. Cell viability representing the mean (+SD) from a total of six determinants is presented relative to that of mock treatment or control subline.

**Figure 5:**
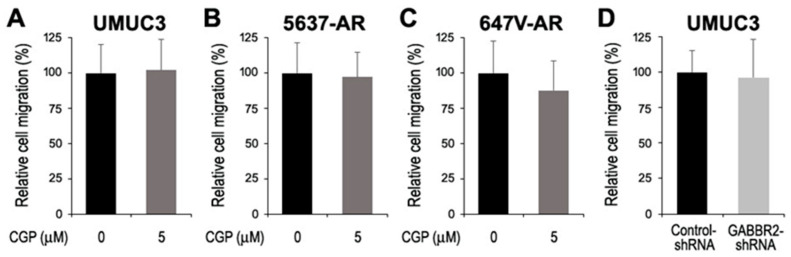
Effects of GABBR2 inhibitor or knockdown on the migration of bladder cancer cells. The wound-healing assay in UMUC3 (**A**), 5637-AR (**B**), or 647V-AR (**C**) without or with CGP46381 (5 μM) or UMUC3-control-shRNA vs. UMUC3-GABBR2-shRNA (**D**) cultured for 24 h after scratching. Cell migration determined by the rate of cells filling the wound area is presented relative to that of mock treatment or control subline. Each value represents the mean (+SD) from a total of six determinants.

Using its inhibitor and knockdown, we assessed the impact of GABBR2 on the growth of bladder cancer cells. Western blot showed that a selective GABA B receptor antagonist, CGP46381 (50% inhibitory concentration (IC50): 4.9 μM [25]), inhibited GABBR2 expression in UMUC3 cells (Figure 3A). Additionally, as expected, the level of GABBR2 expression in a UMUC3 subline stably expressing GABBR2-shRNA was substantially reduced, compared with control-shRNA-expressing cells (Figure 3B). Nonetheless, CGP46381 treatment in three AR-positive bladder cancer cell lines, as well as GABBR2 knockdown in UMUC3 cells, did not significantly change their proliferation (via MTT assay (Figure 4)) or migration (via wound-healing assay (Figure 5)).

### 2.3. Role of GABBR2 in CDDP Sensitivity

**Figure 6:**
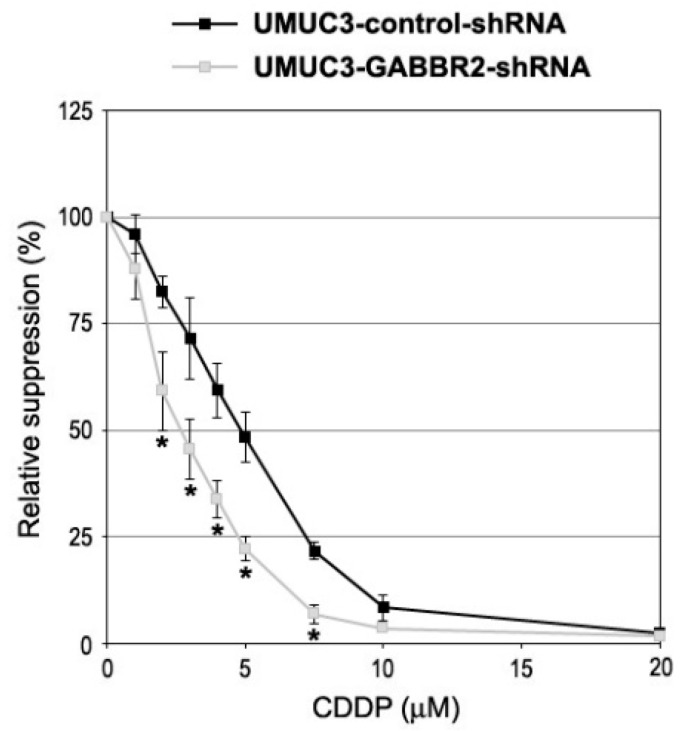
Effects of GABBR2 knockdown on CDDP cytotoxicity in bladder cancer cells. The MTT assay in UMUC3-control-shRNA vs. UMUC3-GABBR2-shRNA sublines cultured for 48 h in the presence of 0–20 µM CDDP. Cell viability representing the mean (±SD) from a total of six determinants is presented relative to that of each subline without CDDP treatment. * *p* < 0.05 (vs. control-shRNA).

We then determined if GABBR2 could modulate sensitivity to CDDP treatment in bladder cancer cells. The MTT assay was first performed in UMUC3-control-shRNA and UMUC3-GABBR2-shRNA sublines treated with various doses of CDDP covering its pharmacological concentrations (i.e., 1.3–8.4 μM [26]). GABBR2-knockdown cells were significantly more sensitive to CDDP at 2–7.5 μM, and the IC50s were 4.8 µM in UMUC3-control-shRNA vs. 2.7 µM in UMUC3-GABBR2-shRNA (Figure 6).

**Figure 7:**
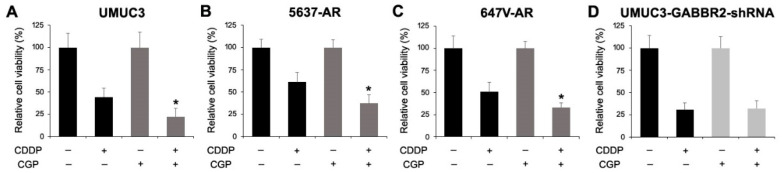
Effects of GABBR2 inhibitor on CDDP cytotoxicity in bladder cancer cells. The MTT assay in UMUC3 (**A**), 5637-AR (**B**), or 647V-AR (**C**) without or with CGP46381 (5 μM) or UMUC3-control-shRNA or UMUC3-GABBR2-shRNA (**D**) cultured for 48 h in the absence or presence of CDDP (5 μM). Cell viability representing the mean (+SD) from a total of six determinants is presented relative to that of mock or CGP46381 treatment (without vs. with CDDP) in each subline. * *p* < 0.05 [vs. CDDP(+)/CGP46381(−) cells].

We further assessed the effects of CGP46381 on CDDP cytotoxicity. In UMUC3 (Figure 7A), 5637-AR (Figure 7B), and 647V-AR (Figure 7C) cells, CGP46381 treatment enhanced the cytotoxic effects of CDDP over mock treatment. However, no significant effects of CGP46381 on CDDP suppression were seen in GABBR2-knockdown cells (Figure 7D).

### 2.4. Prognostic Value of GABBR2 in Bladder Cancer Patients

**Figure 8:**
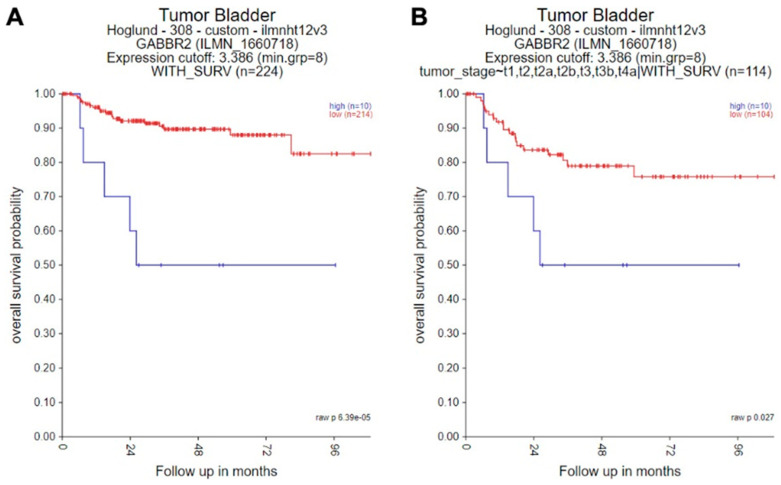
Kaplan–Meier curves for overall survival, according to the levels of *GABBR2* expression in the entire cohort of patients with bladder cancer (**A**) or those with invasive (stage T1 or higher) tumor (**B**).

A publicly available database was searched for analyzing the prognostic significance of *GABBR2* expression in bladder cancer. Data obtained from GSE32894 [27] showed that the rate of overall survival was significantly lower in the entire cohort of patients with high *GABBR2* tumor (Figure 8A). When excluding those with noninvasive tumor who had been much less likely to die of bladder cancer, high *GABBR2* expression was still associated with a significantly higher mortality rate (Figure 8B).

## 3. Discussion

The efficacy of CDDP-based combined systemic chemotherapy in patients with urothelial cancer is often limited due to the initial failure or the acquisition of drug resistance during treatment, for which underlying molecular mechanisms remain not fully understood. Meanwhile, AR activation in bladder cancer cells has been associated with induction of resistance to CDDP [21,22,23,28,29,30] as well as other chemotherapeutic agents [31,32]. In the present study, we further investigated the functional role of GABBR2 in CDDP resistance in bladder cancer in relation to AR signaling.

GABA B receptors, including GABBR1 and GABBR2, are G protein-coupled receptor subunits. As GABA represents the main neurotransmitter in the brain, GABBR1 and/or GABBR2 have been implicated in the pathogenesis of various neurological or psychiatric disorders such as epilepsy, Huntington’s disease, Alzheimer’s disease, autism, and depression [33]. By contrast, little is known about the role of GABBR2 in neoplastic conditions. Limited and somehow conflicting data have demonstrated that GABA B receptor agonists either promote (e.g., breast [34], kidney [35], and prostate [36] cancers) or inhibit (e.g., lung cancer [37], cholangiocarcinoma [38,39]) the cell growth of nonurothelial malignancies. Particularly, in the prostate cancer study [36], an AR-negative cell line was tested, although the role of AR signaling in its progression and treatment has been extremely well established. In other studies, however, GABA B receptor agonists have been shown to have no significant effects on the cell proliferation and/or migration/invasion of colon [40] and liver [41] cancer lines as well as both AR-positive and AR-negative prostate cancer lines [42]. In a more recent study, a microRNA miR-31-3p was shown to suppress the growth of prostate cancer cells via directly downregulating GABBR2 expression similarly in AR-positive and AR-negative lines [43]. Thus, the involvement of GABBR2 in urothelial cancer progression or chemoresistance, as well as that in AR signaling, remains largely unknown.

As aforementioned, it remains unanswered how AR signaling modulates chemosensitivity. Based on the findings in DNA microarray analysis in control AR-positive vs. AR-knockdown bladder cancer sublines we had previously performed [24], we anticipated that GABBR2 functioned as a downstream target of AR. Herein, we demonstrated that AR expression or activity was positively correlated with GABBR2 expression. Specifically, androgen treatment and AR knockdown/antiandrogen treatment induced and reduced, respectively, the levels of GABBR2 expression in bladder cancer cells. Notably, a ChIP assay revealed an interaction of AR with GABBR2 at its promoter region, indicating the direct regulation of GABBR2 expression by AR. We then found that knockdown of GABBR2 via its shRNA or its inactivation via a GABA B receptor antagonist enhanced the cytotoxic effects of CDDP in AR-positive bladder cancer cells. Because the inhibitor failed to induce CDDP sensitivity in GABBR2-knockdown cells, its effect on CDDP cytotoxicity was likely mediated through GABBR2. However, GABBR2 did not appear to affect bladder cancer cell proliferation and migration in the absence of CDDP. These observations suggest that activation of AR induces CDDP resistance via directly upregulating the expression of GABBR2 in bladder cancer cells. Interestingly, in several nonurothelial cancer cells, cooperation of GABBR2 with EGFR-ERK1/2 signaling has been documented [35,36,37,38,44], while the ERK1/2 pathway has been linked to CDDP resistance [29,45]. Meanwhile, we have separately demonstrated that androgens activate the EGFR-ERK1/2 pathway in AR-positive bladder cancer cells [46].

GABBR2 expression has not been extensively studied in clinical samples. Nonetheless, the elevated expression of GABBR2 has been reported in several types of neoplasm such as thyroid adenoma and carcinoma [47] and non-small cell lung cancer [37]. In cholangiocarcinoma specimens, high GABBR2 expression was strongly associated with more aggressive nonpapillary histology yet small tumor size [39]. By analyzing a public database (i.e., The Cancer Genome Atlas (TCGA)), *GABBR2* overexpression was linked to lower T and N staging categories and better prognosis in patients with breast cancer [48]. Similarly, using a publicly available database [27], we here found that those with invasive bladder cancer showing high *GABBR2* expression had a significantly higher risk of overall mortality. This observation indeed supports our in vitro data indicating that GABBR2 could induce CDDP resistance in bladder cancer, although only a portion of patients in the database cohort may have undergone CDDP-based chemotherapy.

Again, in the present study, we have assessed if GABBR2 functions as a downstream effector of AR and, thereby, contributes to modulating sensitivity to CDDP. We first demonstrated that AR directly regulated the expression of GABBR2 via binding to its promoter in bladder cancer cells. AR inactivation, particularly via antiandrogen treatment, thus resulted in downregulation of GABBR2 expression. We then found that GABBR2 was involved in promoting CDDP resistance in bladder cancer cells. An antagonist for GABBR2 at the dose of IC50 thus significantly increased sensitivity to CDDP therapy. These findings suggest that the concurrent treatment with not only an antiandrogen, which has been widely used in patients with, for example, prostate cancer, but also a GABBR2 inhibitor, may considerably enhance the efficacy of CDDP-based chemotherapy, particularly in patients with AR-positive/GABBR2-positive bladder cancer. However, because an available inhibitor, CGP46381, is not entirely specific for GABBR2, the involvement of another GABA B receptor, GABBR1, in modulating CDDP sensitivity may need to be determined. Meanwhile, molecular subtypes of bladder cancer have been well established (e.g., TCGA cohort study [49]) and certain subtypes of muscle-invasive bladder cancer have been associated with chemoresistance [50]. It might, therefore, be of importance to determine which molecular subgroup of patients could generally benefit from such combination therapy.

## 4. Materials and Methods

### 4.1. Antibodies and Chemicals

We obtained anti-AR (441), anti-GABBR2 (H-10), and anti-GAPDH (6c5) antibodies from Santa Cruz Biotechnology (Dallas, TX, USA). DHT and HF, CGP46381, and CDDP were obtained from Sigma-Aldrich (St. Louis, MO, USA), Thermo Fisher (Waltham, MA, USA), and Santa Cruz Biotechnology (Dallas, TX, USA), respectively.

### 4.2. Cell Lines

A human urothelial carcinoma cell line, UMUC3, was originally obtained from the American Type Culture Collection and recently authenticated by the institutional core facility. Human urothelial cancer sublines stably expressing AR-shRNA (e.g., UMUC3-AR-shRNA) or control-shRNA (e.g., UMUC3-control-shRNA) [47], as well as human wild-type AR (e.g., 5637-AR [47], 647V-AR [51]), were established in our previous studies. Similarly, GABBR2-shRNA (sc-42463-V; Santa Cruz Biotechnology), a pool of concentrated, transduction-ready lentiviral particles containing three target-specific constructs encoding 19–25 nt shRNA, was stably expressed in UMUC3 cells. In addition, a CDDP-resistant subline (i.e., UMUC3-CR) and its control were previously established by long-term (i.e., >12 weeks), continuous, stepwise (i.e., 0.2–2.0 μM) treatment of CDDP [21]. These parental line and sublines were maintained in DMEM (Thermo Fisher) supplemented with 10% fetal bovine serum (FBS), penicillin (50 U/mL), and streptomycin (50 μg/mL) at 37 °C in a humidified atmosphere of 5% CO_2_. The cells were then cultured in phenol red-free medium supplemented with 5% FBS (or 5% charcoal-stripped FBS for DHT/HF treatment) at least 24 h before actual assays.

### 4.3. Real-Time PCR

Total RNA isolated from cultured cells by TRIzol (Invitrogen, Waltham, MA, USA) was reverse transcribed using oligo (dT) primers and Omniscript reverse transcriptase (Qiagen, Germantown, MD, USA). Real-time PCR was then performed using iQ™ SYBR^®^ Green Supermix (Bio-Rad, Hercules, CA, USA), as we described previously [21,24]. The following primer pairs were used: *GABBR2* (forward, 5′-TGGAGGCGTCTGTCCATCCGT-3′; reverse, 5′-GTCTTGCGTCAGCGTGCCCA-3′); and *GAPDH* (forward, 5′-AAGGTGAAGGTCGGAGTCAAC-3′; reverse, 5′-GGGGTCATTGATGGCAACAATA-3′).

### 4.4. Western Blot

Equal amounts of proteins (30 µg) extracted from cell extracts were subjected to electrophoresis with 10% sodium dodecyl sulfate-polyacrylamide gel, which was transferred to polyvinylidene difluoride membrane electronically. After blocking with 5% nonfat dry milk, the membrane was incubated with a primary antibody at 4 °C overnight, followed by 1 h incubation with an HRP-conjugated secondary antibody (Cell Signaling Technology, Danvers, MA, USA) at room temperature. Chemiluminescent signals were then generated by Clarity Western ECL Substrate and detected by ChemiDOC™ MP (Bio-Rad).

### 4.5. ChIP

We first performed a bioinformatic search for potential AR binding sites in the GABBR2 promoter (https://biogrid-lasagna.engr.uconn.edu/lasagna_search/ [52], accessed on 2 March 2022) (see Figure 2). ChIP assay was then performed, using a Magna ChIP kit (Sigma-Aldrich), according to the manufacturer’s recommended protocol with minor modifications, as we described previously [30]. Briefly, cells were cross-linked with 1% formaldehyde, and the lysates were sonicated in nuclear buffer. Soluble chromatin was immunoprecipitated with an anti-AR antibody or normal mouse immunoglobulin G (IgG) (Santa Cruz Biochemistry) directly conjugated with magnetic protein A beads. Immunoprecipitated DNA was eluted and reverse cross-linked. DNA was then extracted and purified, using a spin filter column, and amplified by PCR, using the primers as follows: forward, 5′-ACGCCTTCGTGGAACATT-3′; reverse, 5′-ACAGCGATCTGGGAACC-3′. The PCR products electrophoresed on 1% agarose gel and stained with ethidium bromide were visualized, using Gel Doc XR + (Bio-Rad).

### 4.6. Cell Proliferation

The MTT assay was used to assess the cell viability. Cells (5 × 10^3^/well) seeded in 96-well tissue culture plates were cultured for 48 h and then incubated with 0.5 mg/mL of MTT (3-(4,5-dimethylthiazol-2-yl)-2,5diphenyltetrazolium bromide; Sigma-Aldrich) for 3 h at 37 °C. MTT was dissolved by dimethyl sulfoxide, and the absorbance was measured at a wavelength of 570 nm with background subtraction at 630 nm. IC50 was calculated using the web-based tool (https://www.aatbio.com/tools/ic50-calculator, accessed on 7 August 2023).

### 4.7. Cell Migration

Scratch wound healing assay was adapted to evaluate the ability of cell migration. Cells at a density of ≥90% confluence in 6-well tissue culture plates were scratched manually with a sterile 200 μL plastic pipette tip. The wounded monolayers of the cells were allowed to heal in serum-free medium for 24 h, and the width of the wound area was monitored with an inverted microscope. The normalized cell-free area in photographed pictures (24 h/0 h) was quantitated using ImageJ software (National Institutes of Health, Bethesda, MD, USA).

### 4.8. Public Database Analysis

The R2 Genomics Analysis and Visualization Platform (https://hgserver1.amc.nl/cgi-bin/r2/main.cgi, accessed on 7 August 2023) was used to assess the prognostic role of *GABBR2* expression in patients with bladder cancer. Data were obtained from the Gene Expression Omnibus repository (GSE32894; https://www.ncbi.nlm.nih.gov/geo/query/acc.cgi?acc=GSE32894 [27], accessed on 7 August 2023).

### 4.9. Statistical Analysis

Student’s *t*-test was used to compare numerical data. *p* values less than 0.05 were considered to be statistically significant.

## 5. Conclusions

We identified GABBR2 as a key downstream effector of AR in modulating CDDP sensitivity in bladder cancer. Our data suggest that not only concurrent antiandrogen therapy but also GABBR2 inhibitor treatment has the potential of being a means of chemosensitization, especially in patients with AR/GABBR2-positive bladder tumor. Nonetheless, further studies are warranted for elucidating molecular mechanisms responsible for AR/GABBR2-mediated chemoresistance.

## Data Availability

The data presented in this study are available on request from the corresponding author but are not publicly available (except public database analysis data—Section 2.4).
